# Supplementary material for: Identifying ITGB2 as a Potential Prognostic Biomarker in Ovarian Cancer
Source: Diagnostics (Basel). 2023 Mar 18;13(6):1169. doi: 10.3390/diagnostics13061169 (PMC10047357; doi:10.3390/diagnostics13061169)
Supplement: Supplementary file 1 [file diagnostics-13-01169-s001.zip › diagnostics-2245084-supplementary.pdf]

Supporting information

GO	Category	Description	Count	%	Log10(P)
GO:0030155	GO Biological Processes	regulation of cell adhesion	42	12.8	-16.73
GO:0098609	GO Biological Processes	cell-cell adhesion	29	8.84	-11.75
GO:0034330	GO Biological Processes	cell junction organization	27	8.23	-11.4
GO:0035239	GO Biological Processes	tube morphogenesis	31	9.45	-10.92
hsa05150	KEGG Pathway	Staphylococcus aureus infection	13	3.96	-10.38
GO:0007162	GO Biological Processes	negative regulation of cell adhesion	20	6.1	-9.72
WP5087	WikiPathways	Malignant pleural mesothelioma	23	7.01	-9.02
R-HSA-1474244	Reactome Gene Sets	Extracellular matrix organization	19	5.79	-8.99
WP2877	WikiPathways	Vitamin D receptor pathway	14	4.27	-7.73
GO:0030855	GO Biological Processes	epithelial cell differentiation	24	7.32	-7.6
hsa05165	KEGG Pathway	Human papillomavirus infection	18	5.49	-7.54
R-HSA-913531	Reactome Gene Sets	Interferon Signaling	14	4.27	-7.36
GO:0006935	GO Biological Processes	chemotaxis	22	6.71	-7.33
GO:0048729	GO Biological Processes	tissue morphogenesis	23	7.01	-7.24
GO:0002683	GO Biological Processes	negative regulation of immune system process	20	6.1	-7.19
GO:0007229	GO Biological Processes	integrin-mediated signaling pathway	10	3.05	-7.14
R-HSA-9006934	Reactome Gene Sets	Signaling by Receptor Tyrosine Kinases	22	6.71	-7.11
GO:0001667	GO Biological Processes	ameboidal-type cell migration	13	3.96	-6.87
GO:0050878	GO Biological Processes	regulation of body fluid levels	18	5.49	-6.8
R-HSA-1280215	Reactome Gene Sets	Cytokine Signaling in Immune system	25	7.62	-6.45

Table S1 Top 20 significantly enriched GO terms and biological functional pathways of DEGs. DEGs were used as the enrichment background. Top 20 clusters with their representative enriched terms (one per cluster) were shown in the table. "Count" is the number of genes in the user-provided lists with membership in the given ontology term. "%" is the percentage of all of the user-provided genes that are found in the given ontology term (only input genes with at least one ontology term annotation are included in the calculation). "Log10 (P) " is the p-value in log base 10.

Rank	Name	Gene description	Score	P
1	CDH1	cadherin 1, type 1, E-cadherin (epithelial)	116	0.0987
2	VEGFA	vascular endothelial growth factor A	92	1.80E-05(***)
3	EPCAM	epithelial cell adhesion molecule	68	0.0312
4	ITGB2	integrin, beta 2 (complement component 3 receptor 3 and 4 subunit)	54	0.0027(*)
5	CLDN7	claudin 7	50	0.0118
6	MUC1	mucin 1, cell surface associated	46	0.1571
7	CLDN4	claudin 4	44	0.0047(*)
8	NANOG	Nanog homeobox	42	0.0664
9	OCLN	occludin	40	9.10E-06(***)
10	CDKN2A	cyclin-dependent kinase inhibitor 2A	38	0.1159
10	LYN	LYN proto-oncogene, Src family tyrosine kinase	38	0.2447
10	SPP1	secreted phosphoprotein 1	38	7.70E-07(***)

Table S2 Top ten in network string interactions ranked by degree method and their p value of progression free survival analysis. (*P<0.01,**P<0.001,***P<0.0001)

Description	ITGB2		SPP1		CLDN4		OCLN		VEGFA	
	R	P	R	P	R	P	R	P	R	P
CD4+TCell	0.396846047	***	0.208301315	***	-0.023080941	0.613961473	-0.058929655	0.197454379	0.019349882	0.672390878
CD8+TCell	0.37536689	***	0.143292648	*	0.137658164	*	0.0477127	0.296854933	-0.136287772	*
B cells	0.239343309	***	0.048491643	0.289029742	0.067714768	0.138502973	0.072070532	0.114813813	-0.124455119	*

Macrophage	0.287306536	***	0.268477429	***	0.072352991	0.113394795	-0.007239379	0.87430284	-0.279890959	***
MacrophageM0	0.157	0.0132	0.244	**	−0.097	0 . 125	0.018	0.772	0.356	***
MacrophageM1	0.353	***	0.181	*	0.005	0.944	0.056	0.375	0.063	0.322
MacrophageM2	0.707	***	0.421	***	0.012	0.854	−0.013	0.833	0.029	0.649

Table S3 Correlation between CLDN4, OCLN, SPP1, and VEGFA expression and the level of immune infiltration (*P<0.01,**P<0.001,***P<0.0001).

Description	Gene markers	ITGB2		SPP1		CLDN4		OCLN		VEGFA	
		R	P	R	P	R	P	R	P	R	P
TAM	CCL2	0.4	***	0.42	***	0.041	0.4	-0.038	0.43	0.089	0.067
	CCL5	0.51	***	0.25	***	0.044	0.36	-0.056	0.25	0.015	0.75
	CD68	0.84	***	0.69	***	0.071	0.14	0.063	0.2	0.13	*
	IL10	0.5	***	0.43	***	0.12	0.012	0.074	0.13	0.13	*
M1 Macrophage	CXCL10	0.26	***	0.21	***	0.058	0.23	0.005	0.92	0.098	0.044
	TNF	0.27	***	0.25	***	0.2	***	0.046	0.34	0.21	***
	INOS（NOS2）	-0.0015	0.98	-0.043	0.38	-0.043	0.37	-0.04	0.41	-0.0083	0.86
	IRF5	0.44	***	0.32	***	0.27	***	0.19	***	0.2	***
	CD80	0.53	***	0.45	***	0.068	0.16	0.13	*	0.22	***
M2 Macrophage	CD163	0.68	***	0.59	***	0.066	0.17	-0.066	0.17	0.066	0.17
	VSIG4	0.75	***	0.65	***	0.066	0.18	-0.02	0.69	0.062	0.2
	MS4A4A	0.72	***	0.51	***	-0.0096	0.84	-0.0033	0.95	0.067	0.17
	CD206（MRC1）	0.56	***	0.42	***	-0.043	0.38	0.036	0.46	0.1	0.04

Table S4 Correlation analysis between ITGB2, SPP1, CLDN4, OCLN, and VEGFA and markers of macrophages in GEPIA database (*P<0.01,**P<0.001,***P<0.0001).

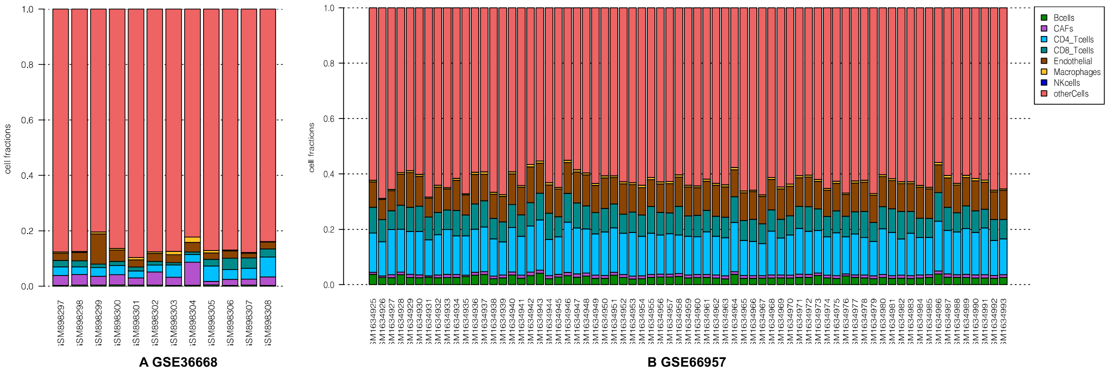

Figure S1 The proportion of immune cells such as CAFs, CD4^{+}/ CD8^{+}Tcells, endothelial, macrophages, NK cells, and other cells in the GSE36668 and GSE66957 datasets.

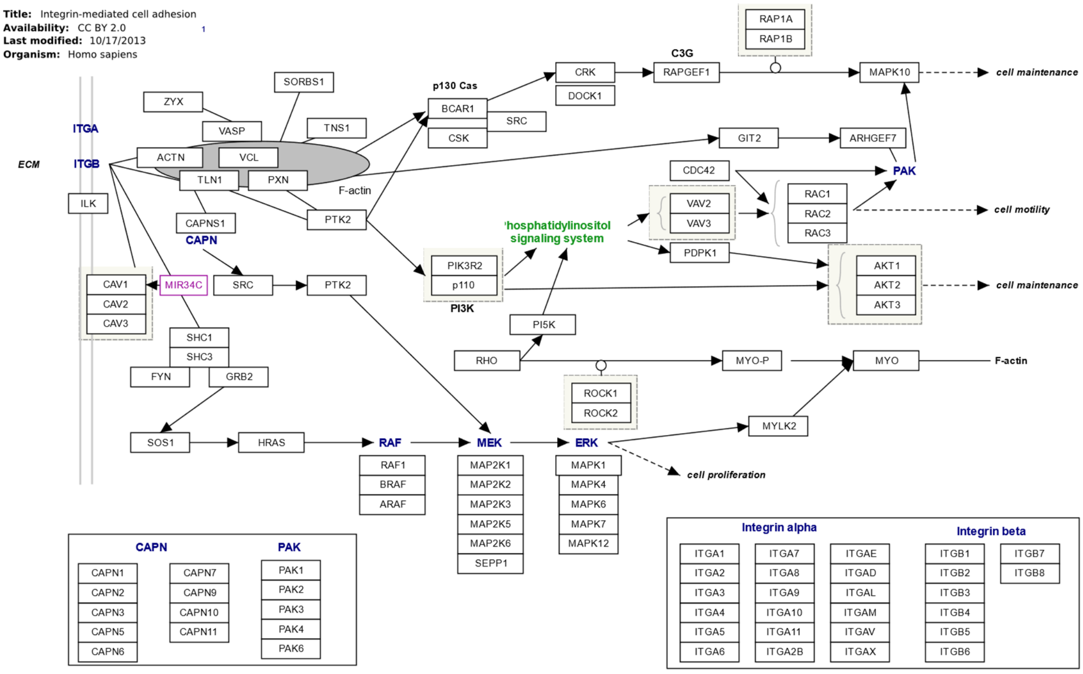

Figure S2 Integrin-mediated cell adhesion pathway.

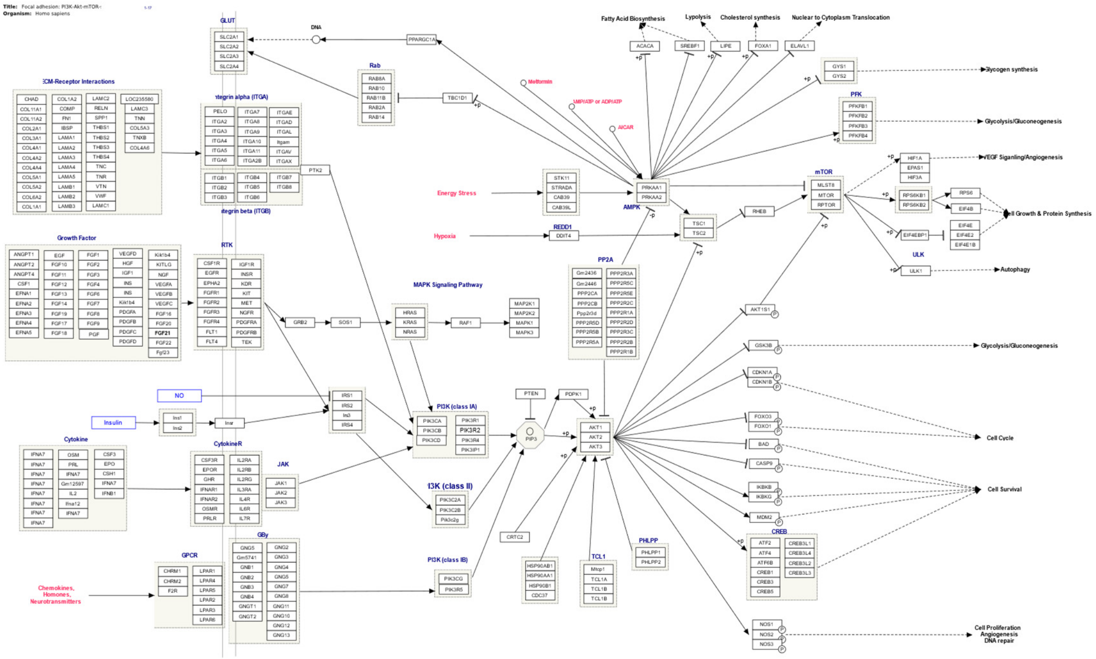

Figure S3 Focal adhesion: PI3K-Akt-mTOR-signaling pathway.
